# On the Removal of the Placenta

**Published:** 1883-12-20

**Authors:** 


### ON THE REMOVAL OF THE PLACENTA.

Prof. Dohm (Deutsche Med. Wochenschrift) gives the result of his observations in the Konigsberg Hospital, especially with regard to Crede's method, as follows:

1. In 1,000 cases of labor where the removal of the placenta was left to nature, the results were far better than in 1,000 other cases where Crede's method was employed.

2. The 1,000 cases of labor where the placenta was discharged spontaneously had markedly less hemorrhage, retention of membranes and puerperal fever. Those that were treated according to Crede's method suffered to a considerable extent from troubles with the membranes, and in consequence there were many fatal puerperal affections.

3. Those cases where the placenta was removed in the first five minutes after birth by the Crede method, were the most liable to these affections. Those that were left longer before such extraction was attempted did better, but still remained considerably in excess of those where this was left to nature.—*Jour. Med. Association.*

[Thus we see that "medlesome midwifery is bad." There is too much interference with the processes of nature in these times. Too much is attempted, not only in obstetric practice, but in all other departments. Patience, prudence and common sense are highly important elements in the make-up of a good practitioner. —ED.] W.

